# Where have all the susceptible gonococci gone? A historical review of changes in MIC distribution over the past 75 years

**DOI:** 10.1186/s12879-019-4712-x

**Published:** 2019-12-27

**Authors:** Chris Kenyon, Jolein Laumen, Dorien Van Den Bossche, Christophe Van Dijck

**Affiliations:** 1 HIV/STI Unit, Institute of Tropical Medicine, Antwerp, Belgium; 2 Division of Infectious Diseases and HIV Medicine, University of Cape Town, Anzio Road, Observatory, Cape Town, 7700 South Africa

**Keywords:** *Neisseria gonorrhoeae*, Gonococcus, Antimicrobial resistance, AMR, MIC, MIC-shift, ECOFF

## Abstract

**Background:**

Does the emergence of antimicrobial resistance in *Neisseria gonorrhoeae* include the erasure of highly susceptible strains or does it merely involve a stretching of the MIC distribution? If it was the former this would be important to know as it would increase the probability that the loss of susceptibility is irreversible.

**Methods:**

We conducted a historical analysis based on a literature review of changes of *N. gonorrhoeae* MIC distribution over the past 75 years for 3 antimicrobials (benzylpenicillin, ceftriaxone and azithromycin) in five countries (Denmark, Japan, South Africa, the United Kingdom and the United States).

**Results:**

Changes in MIC distribution were most marked for benzylpenicillin and showed evidence of a right shifting of MIC distribution that was associated with a reduction/elimination of susceptible strains in all countries. In the case of ceftriaxone and azithromycin, where only more recent data was available, right shifting was also found in all countries but the extent of right shifting varied and the evidence for the elimination of susceptible strains was more mixed.

**Conclusions:**

The finding of right shifting of MIC distribution combined with reduction/elimination of susceptible strains is of concern since it suggests that this shifting may not be reversible. Since excess antimicrobial consumption is likely to be responsible for this right shifting, this insight provides additional impetus to promote antimicrobial stewardship.

## Background

**Fig. 1:**
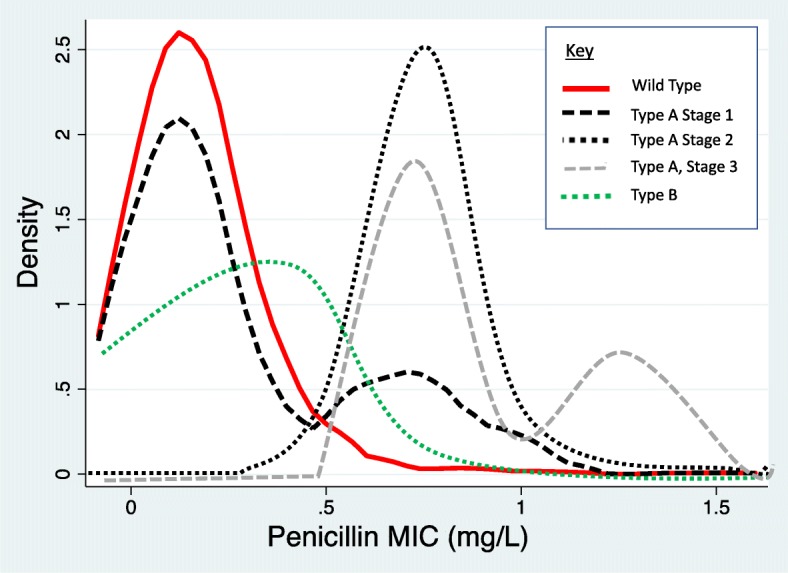
A schematic representation of how *N. gonorrhoeae* benzylpenicillin MIC distributions could change over time. A type A shift involves in the first stage, the emergence of a second population of less susceptible isolates (black, long-dashed line). With further selection pressure all the gonococci move into the higher MIC peak resulting in a right-shifted unimodal distribution (type A, stage 2, black, short-dashed line). This distribution could then develop a new subpopulation with higher MICs resulting in a new bimodal distribution (Type A, stage 3; grey, long-dashed line). Alternatively, the decrease in susceptibility results from a shift in the whole population (type B shift, green, short-dashed line)

Typically, investigations of antimicrobial resistance (AMR) in *Neisseria gonorrhoeae* have focused on the right-hand tail of the MIC distributions (the subpopulation of *N. gonorrhoeae* that acquires AMR) [1–3]. An under investigated topic is what happens to the left-hand tail of the MIC distribution and whether or not the emergence of AMR is associated with a right shifting of the entire MIC distribution including the left-hand tail [4–7]? One previous ecological analysis of 5 year data from 24 European countries found that the emergence of gonococcal AMR was associated with a right shift of the susceptible isolates [6]. The study did not however characterize the nature of the right shift. Right shifting could occur via a number of pathways. Firstly, if a subpopulation of *N. gonorrhoeae* acquires AMR this could lead to a bimodal distribution in the MIC with no/little change in the MIC of the original ‘wild type’ susceptible population (Fig. 1, type A, stage 1) [6]. In a second stage of this process and following further selection pressure, the most susceptible gonococci could become rare. The new ‘wild type’ gonococci would now constitute a new MIC peak with a right-shifted unimodal distribution (type A, stage 2). This distribution could then develop a new subpopulation with even higher MICs resulting in a new bimodal distribution (Type A, stage 3 etc.). Secondly, the acquisition of AMR could be followed by a zero-sum stretching of the entire MIC distribution where the isolates in the left-hand tail are reduced in direct proportion to the increase of isolates in the right-hand tail (Fig. 1, type B). Thus in a type A stage 1 shift, the increase in gonococcal AMR leads to a wider MIC distribution. This is then followed, in the second stage, by a reduction in MIC distribution width as most of the highly susceptible isolates are lost through evolution. This process leads to the (close to) elimination of the highly susceptible isolates in type A (stage 2 and 3) but not B shifts.

An elimination of highly susceptible strains would likely increase the risk that the loss of susceptibility is irreversible. This may have important consequences for not only gonococcal AMR but also that of commensal bacteria. There is increasing evidence that high antimicrobial exposure increases the risk of AMR in *N. gonorrohoeae* as well as various other *Neisseriae* and other commensal species [6–9]. If excess antimicrobial consumption results in a type A, stage 2 or 3 trajectory in not only *N. gonorrhoeae* but also in these commensal species then this may be a warning of more wide-ranging adverse effects of antimicrobials on our microbiomes and resistomes [10].

In this review we conducted a non-quantitative historical analysis of how *N. gonorrhoeae* MIC distributions for 3 antimicrobials (benzylpenicillin, ceftriaxone and azithromycin) have changed over time in five countries.

## Methods

### Data

#### Historical review

We selected the five countries, Denmark, Japan, South Africa, the United Kingdom (UK) and the United States (USA) for the study based on the availability and quality of data over a long enough time period to answer our study question. Further selection considerations included trying to ensure a mix of regions or countries with the best quality data from early on in the antibiotic era (Denmark, the UK and the USA). We included Japan since this country has been noted to be among the first where AMR has arisen on a number of occasions [2, 11]. Whilst gonorrhoea transmission disproportionately affects core-groups in these three high income countries, this is not the case in South Africa which was chosen to represent a low to middle income country where most transmission has been in the general heterosexual population [12].

### Search strategy and selection criteria

We conducted a literature review using PUBMED and Google Scholar to find studies reporting minimum inhibitory concentrations (MIC) or 50% inhibitory concentration (IC50) distributions from 1940 to the present. MeSH terms used in the PubMed search (search date: 10/03/2019) were “Denmark” OR “Japan” OR “South Africa”, OR “United States” OR “United Kingdom” OR “England” OR “Scotland” OR “Wales” OR “Ireland”; AND: “gonorrhoea” OR “gonorrhea” OR “*Neisseria gonorrhoeae*”; filters: publication date from 01/01/1940 until 09/03/2019. Similar search terms were used for the Google Scholar search (search date: 30/03/2019). In addition we searched national surveillance websites for AMR reports: Denmark (www.ssi.dk), Japan (www.niid.go.jp), South Africa (http://www.nicd.ac.za), United Kingdom (https://www.gov.uk/government/publications/gonococcal-resistance-to-antimicrobials-surveillance-programme-grasp-report) and the United States (https://www.cdc.gov/std/gisp/default.htm). No studies from the Republic of Ireland were included.

### Inclusion and exclusion criteria

Studies were eligible for inclusion if they reported the IC50 or MIC distributions for any of the three antibiotics and the following was true:

The sampling strategy was not overtly biased. Samples needed to either be from all or a non-selected group of patients attending primary care level STI clinics, or based on national AMR surveillance reports. We excluded all studies which reported samples where higher AMR was probable – such as samples which had suspected AMR sent for confirmatory testing.

We selected the studies reporting MIC distributions from isolates at the earliest and latest dates from each country for each antimicrobial. In addition where ever possible we selected a MIC distribution from the midpoint between these two time points. All data are presented as MICs or IC50s. The 1945 benzylpenicillin susceptibility data from the USA are reported in Oxford units of benzylpenicillin per ml. We multiplied this by 1667 to convert Oxford units to mg of benzylpenicillin [13].

Contemporary EUCAST breakpoints were used to define AMR: azithromycin resistance, > 0.5 mg/L; ceftriaxone resistance, > 0.125 mg/L; and benzylpenicillin resistance, > 1 mg/L (available at: http://www.eucast.org).

### Comparisons with EUCAST’s wild type MIC distributions

We also compare the MIC distributions of the three antimicrobials with those from the European Committee on Antimicrobial Susceptibility Testing (EUCAST) “Antimicrobial wild type distributions of microorganisms” collection [14]. EUCAST has used this data to establish epidemiological cut-offs (ECOFFs) for ceftriaxone (0.032 mg/L) and azithromycin (1 mg/L), but not for benzylpenicillin [14, 15]. For these visual comparisons we compare the EUCAST MIC distributions with the earliest country distribution.

#### Statistical analysis

All analyses were performed in STATA 13.0 (StataCorp LP, College Station, TX, USA). When exact figures were not reported, IC50/MIC distributions were digitalized using GetData Graph Digitizer 2.26. For graphical representation of MIC distributions, if a study reported the lowest MIC distribution as less than or equal to a particular MIC then this MIC value was used.

## Results

### Historical review

**Fig. 2:**
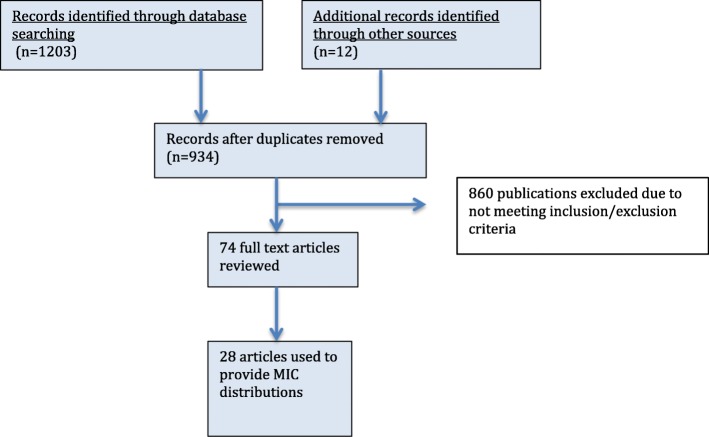
PRISMA flow chart showing selection of publications from the literature search

A total of 1203 articles were identified, and 74 articles reviewed (Fig. 2). Twenty-eight publications were selected to provide the MIC distributions. A summary of the study characteristics used to provide these distributions is given in Table 1.

**Table 1:** Sources and study methodology of benzylpenicillin, ceftriaxone and azithromycin MIC data used in the study

Country/ EUCAST	Antimicrobial	Sampling period	Study design, testing modality and study reference	References
Denmark	Benzylpenicillin	1944 & 1957	1944: 90 strains from 90 non-selected patients stored as lyophilized cultures and tested in 1957 in parallel with and using the same methodology as the 1957 samples as detailed below. 1957: 103 isolates from 96 randomly-selected patients. Benzylpenicillin susceptibility was assessed using the plate dilution and tablet methods. The two methods provided highly concordant results. Results are reported as 50% inhibitory concentrations for the plate dilution method which provided the most reproducible results. The 50% inhibitory concentrations were calculated by means of the Karber method.	[16]
Japan	Benzylpenicillin	1968	33 gonococcal isolates from 33 consecutive navy personnel attending a USA navy STI clinic at Yokosuya, Japan, with a diagnosis of urethritis. MICs were determined via agar dilution following CLSI guidelines.	[17]
		2009–2010	83 isolates from 83 individuals with gonococcal male urethritis presenting to one of 51 facilities in Japan between April 2009 and October 2010 were sent to a central laboratory for benzylpenicillin MIC testing via agar dilution in accordance with CLSI guidelines.	[18]
	Ceftriaxone	1995–2005	MICs were determined using an agar dilution method with a GC agar base containing 1% defined growth supplement. Performed on 690 clinical isolates of *N. gonorrhoeae* obtained from local STI clinics between 1995 and 2005 in Kanagwa, Japan.	[19]
		2010–2013	MICs were determined using an agar dilution method with a GC agar base containing 1% defined growth supplement. Performed on 677 clinical isolates of *N. gonorrhoeae* obtained from the local STI clinic between January 2010 to December 2013 in Fukuoka, Japan.	[3]
	Azithromycin	1981 to 1984 and 1992 to 1993	27 isolates from 1981 to 1984 and 79 isolates from 1992 to 1993 had their MICs determined simultaneously via the same methodology via an agar dilution method with a GC agar base containing 1% defined growth supplement.	[20]
		2013	137 isolates from consecutive male and female patients attending STI clinics in Fukuoka, with ‘genital gonorrhoea’. Susceptibility testing was performed via agar dilution with GC agar.	[3].
		2014/2015	Study performed on 60 isolates from 2014 and 54 isolates from 2015. All isolates were from males with a confirmed case of gonococcal urethritis. Azithromycin MICs were determined via the agar dilution method using GC agar base media supplemented with 1% IsoVitaleX.	[11]
South Africa	Benzylpenicillin	1976–77	175 isolates from non-selected men with urethritis presenting to a GUM clinic in Johannesburg between 1976 and 1977. MICs tested via agar diffusion using Oxoid DST agar.	[20] [18]
		2013	319 isolates from men with urethritis and women with vaginal discharge syndrome presenting to two GUM clinics in Durban and Pietermaritzburg. MICs were tested using the agar dilution method. MICs tested via agar diffusion using Oxoid DST agar.	[21]
	Azithromycin	1999	56 isolates from men with urethritis and women with vaginal discharge syndrome presenting to a single GUM clinic in Durban. MIC testing was performed with agar diffusion using Oxoid DST agar.	[22]
		2013	319 isolates from men with urethritis and women with vaginal discharge syndrome presenting to two GUM clinics in Durban and Pietermaritzburg. MICs were tested using the agar dilution method. MICs tested via agar diffusion using Oxoid DST agar.	[21]
	Ceftriaxone	1995 & 1999	In 1995, 61 isolates and in 1999 58 isolates from men with urethritis and women with vaginal discharge syndrome presenting to a single GUM clinic in Durban. MIC testing was performed with agar diffusion using Oxoid DST agar.	[22]
		2013	319 isolates from men with urethritis and women with vaginal discharge syndrome presenting to two GUM clinics in Durban and Pietermaritzburg. MICs were tested using the agar dilution method. MICs tested via agar diffusion using Oxoid DST agar.	[21]
UK	Benzylpenicillin	1957	Taken from a report produced by the Medical Research Council Working Party to Examine the Resistance of Gonococci to Benzylpenicillin. Results for 302 isolates taken in the London area in April to December 1957 are provided. All samples were tested centrally via the same agar diffusion technique.	[23]
		1994	Isolates were from 113 consecutive patients attending the GUM clinic at an East London GUM clinic over a one-year period. 66% were from men and 3.5% MSM. MICs were determined by agar diffusion.	[24]
		2001	In the 2001 Gonococcal resistance to antimicrobials surveillance programme (GRASP) report, 2542 isolates from 24 participating GUM clinics had their MICs ascertained via agar diffusion using Oxoid DST agar.	[25]
	Ceftriaxone	2003, 2008 and 2015	2003 results are taken from Gonococcal resistance to antimicrobials surveillance programme (GRASP) Report 2003. In this survey 1977 non-duplicate isolates had MIC testing performed with agar diffusion using Oxoid DST agar. Results for 2008 and 2015 are taken from the 2016 Gonococcal resistance to antimicrobials surveillance programme (GRASP) report [26]. **2008 data:** 1276 isolates were tested from 26 GUM clinics in England and Wales. MIC testing performed with agar diffusion using Oxoid DST agar. No further details provided. **2015 data:** 2302 isolates from 1699 unique patients from 23 English GUM clinics were tested. 87% were men and 72% were MSM. Slight over-representation of MSM and London residents. MIC testing performed with agar diffusion using HiMedia DST agar.	[26–28]
	Azithromycin	2001	In the 2001 Gonococcal resistance to antimicrobials surveillance programme (GRASP) report, 2542 isolates from 24 participating GUM clinics had their MICs ascertained via agar diffusion using Oxoid DST agar. 71% were men and 25% MSM.	[25]
		2003	2003 results are taken from Gonococcal resistance to antimicrobials surveillance programme (GRASP) Report 2003. In this survey 1977 non-duplicate isolates had MIC testing performed with agar diffusion using Oxoid DST agar. 72% were from men and 24% from MSM. 47.1% were from London and 52.9% from outside London. Azithromycin MIC distributions for the whole country were not provided but distributions for both London and non-London populations were provided. These were similar and we use the data for the non-London sample as this was larger.	[28]
		2015	2302 isolates from 1699 unique patients from 23 English GUM clinics were tested. 87% were men and 72% were MSM. Slight over-representation of MSM and London residents. MIC testing performed with agar diffusion using HiMedia DST agar	[26]
USA	Benzylpenicillin	1945	104 isolates from a non-selected group of female patients with a diagnosis of gonorrhoea attending a clinic at the University of Texas. MIC were tested via agar dilution on 0.5% starch agar.	[29]
		Pre-1947 and 1949	A study from a single laboratory in Boston that used the same MIC methodology (agar dilution) to test susceptibility for gonococcal isolates obtained before 1947 (*n* = 24) with those from 1949 (*n* = 52). Isolates were from men and women. Heart-infusion agar was used in all assessments.	[30]
	Benzylpenicillin, azithromycin & ceftriaxone	1987/1988, 1992, 2013 & 2017	The typical annual Gonococcal Isolate Surveillance Project (GISP) methodology is as follows: Isolates are collected monthly from up to the first 25 men with N. gonorrhoeae urethritis attending participating STD clinics. Isolates are sent to the regional laboratories for agar dilution antimicrobial susceptibility testing. **GISP 1987** (benzylpenicillin/ceftriaxone): 6204 isolates from men with urethritis from 21 clinics presenting between September 1987 and December 1988. MICs were ascertained at 4 central laboratories using the agar-dilution technique with GC-II Base medium [31]. **GISP 1992** (Azithromycin) Susceptibility testing for azithromycin in GISP began in 1992. GISP 1992 tested 5238 gonococcal isolates [32, 33] **GISP 2013:** (ceftriaxone/azithromicin): In GISP 2017 STD clinics affiliated with 27 state or city health departments sent 5093 gonococcal isolates to GISP. **GISP 2017** (ceftriaxone/azithromicin): In GISP 2017 STD clinics affiliated with 27 state or city health departments contributed 5061 gonococcal isolates to GISP.	[34]
EUCAST collections	Azithromycin	–	3727 isolates from 10 collections	[14]
	Benzylpenicillin	–	11,826 isolates from 21 collections	[14]
	Ceftriaxone	–	1697 isolates from 11 collections	[14]

Abbreviations: GISP - Gonococcal Isolate Surveillance Project; GRASP - Gonococcal resistance to antimicrobials surveillance programme; GUM - GenitoUrinary Medicine Clinic; MSM -Men who have sex with men; STD -Sexually Transmitted Diseases

### Benzylpenicillin

#### Denmark

**Fig. 3:**
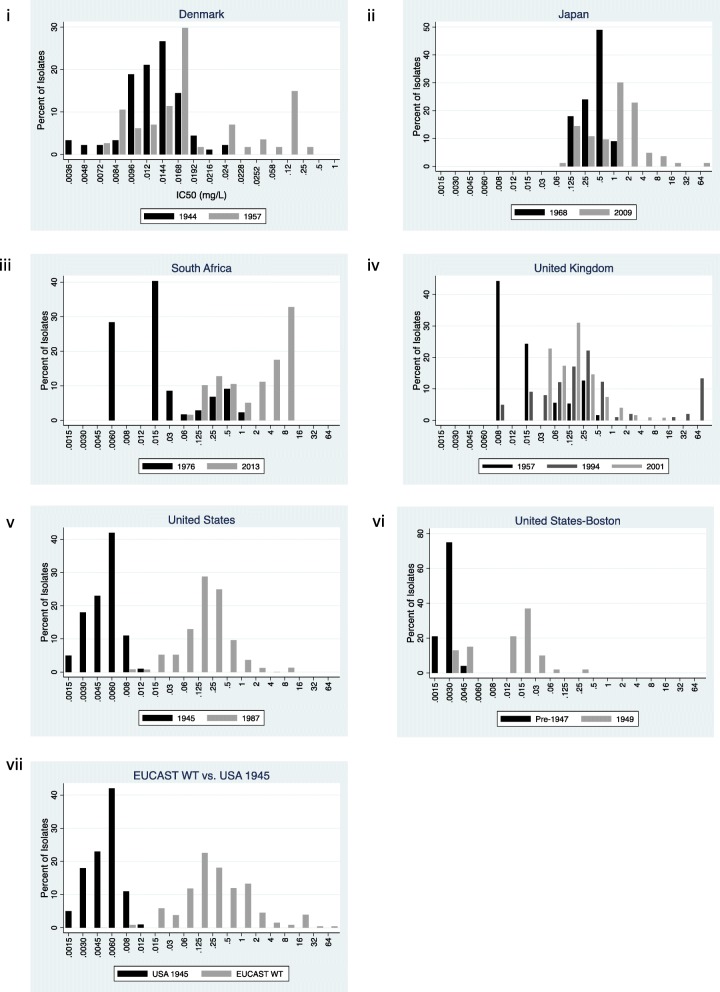
Distribution of *Neisseria gonorrhoeae* benzylpenicillin 50% inhibitory concentrations (IC50) distributions for Denmark (i) and minimum inhibitory concentrations (MIC) distributions for Japan (ii), South Africa (iii), United Kingdom (iv), United States (v), Boston (United States) (vi), and EUCAST wild type collection (EUCAST WT) (vii). All MIC values are reported in mg/L. Please note that the x-axis between 0.015 and 64 mg/L uses a doubling scale and below 0.015 mg/L a less than doubling scale to be able to more accurately represent the distribution of more susceptible strains in the earlier studies

A study by Reyn et al., utilized the same methodology to assess the IC50s of local isolates from 1944 and 1957, in parallel [16]. The IC50 distribution in 1944 was normally distributed with a mode of 0.0144 mg/L. By 1957 the distribution was bimodal with a first peak that was largely unchanged from 1944 and a second peak centered around an IC50 of 0.2 mg/L (Fig. 3).

We could not find papers published after this date that reported benzylpenicillin IC50 or MIC distributions. A Danish surveillance report from 2001 however reported that 40% of 334 isolates in 1999 were benzylpenicillin resistant (MIC > 1 mg/L) and that this figure was unchanged for 5 years [35]. Euro-GASP does not report MIC distributions per country and thus we were unable to obtain this data from this source.

#### Japan

Between 1968 and 2009 the MIC range increased from 0.125–1 to 0.06–64 mg/L [17, 18]. The lowest dilutions tested were 0.125 in 1968 and 0.06 mg/L in 2009. The modal MIC increased from 0.5 to 1 mg/L (Fig. 3).

#### South Africa

The earliest benzylpenicillin MIC distribution was from 1976 and was not dissimilar to the Denmark bimodal distribution in 1957 with peaks at 0.01 and 0.5 mg/L (range 0.006 to 1 mg/L) [36]. By 2013, the bimodal MIC distribution was retained but right shifted with two peaks at 0.25 and 8 mg/L [21]. Only 1.6% of isolates in 2013 had a MIC ≤0.06 mg/L. Less than half (38.6%) of isolates from 2013 had MICs that overlapped with those from 1976 (Fig. 3).

#### UK

In 1957 the benzylpenicillin MIC was approximately bimodally distributed, with peaks at 0.008 (the modal value) and 0.25 mg/L [23]. By 2001, the range had increased from ≤0.008–0.5 to ≤0.03–8 with a right shifted modal value of 0.125 mg/L. The lowest dilutions tested were 0.008 in 1957 and 0.03 mg/L in 2001. The MIC distribution from the midpoint between these two time points was from 1994 and was somewhat right shifted compared to that from 2001. The 2001 data were provided by the UKs’ national surveillance Gonococcal resistance to antimicrobials surveillance programme (GRASP, [25]). Unfortunately, GRASP did not report the benzylpenicillin MIC distribution after 2001.

#### USA

Between 1945 and 1987, the benzylpenicillin MIC distribution shifted from 0.0015–0.12 to 0.008–8 mg/L [29, 31]. Only 1.5% of the 1987 distribution overlapped with the 1945 distribution. The mode increased from 0.006 to 0.125 mg/L. A study from a single laboratory in Boston that used the same MIC methodology (agar dilution) to test susceptibility for gonococcal isolates obtained before 1947 with those from 1949, found an increase in the mode from 0.003 to 0.02 and a right shifting of the distribution from 0.001–0.005 to 0.003–0.2 mg/L (Fig. 3) [30].

#### EUCAST

The EUCAST wild type penicillin MIC distribution was so right shifted compared to the distribution from 1945 in the USA that there is almost no overlap in their distributions (Fig. 3). The 1945 distribution has a mode of 0.006 mg/L and a range up to 0.012 mg/L. The corresponding values for the EUCAST distribution are 0.125 mg/L and 64 mg/L.

### Azithromycin

#### Japan

**Fig. 4:**
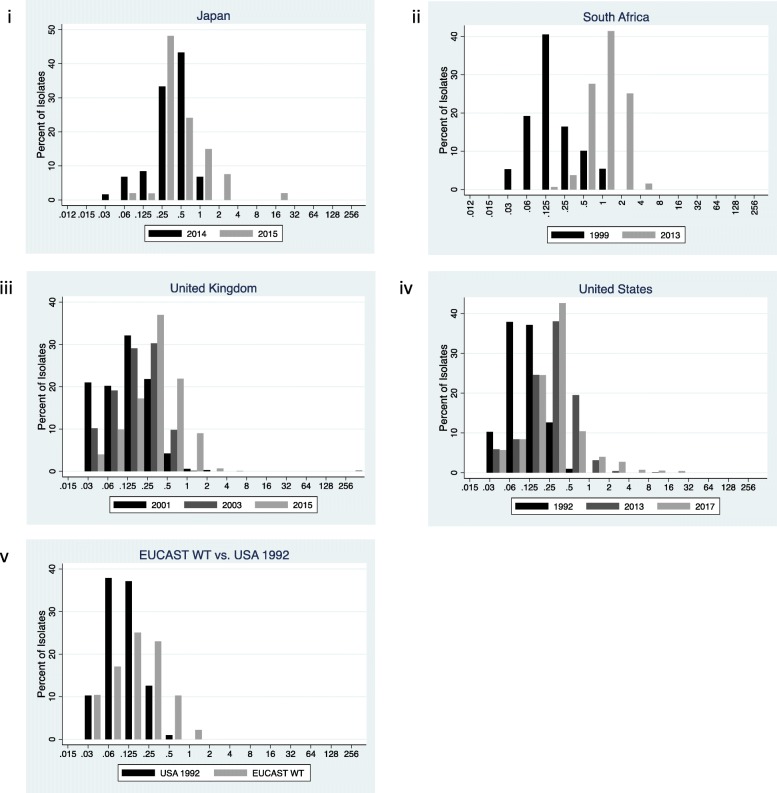
Distribution of *Neisseria gonorrhoeae* azithromycin minimum inhibitory concentrations (MIC) distributions for Japan (**i**), South Africa (**ii**), United Kingdom (**iii**) and the United States (**vi**) and EUCAST wild type collection (EUCAST WT) (**v**). All MIC values are reported in mg/L.

The azithromycin MIC range increased from 0.016–1 mg/L in 1992/93 to 0.015–2 mg/L in 2013 [3, 20]. The MIC50 increased from 0.125 to 0.25 mg/L over this time period. In a two-year period, a study from Sendai, Japan, found a slight right shifting of the MIC distribution between 2014 and 2015, with the range increasing from 0.03–1 to 0.06–16 mg/L (Fig. 4) [11].

#### South Africa

The prevalence of azithromycin resistance increased from 5.4 to 68.0% between 1999 and 2013 in the same clinic in South Africa [21, 22]. This was associated with a right shift of the entire MIC distribution. The modal value increased from a MIC of 0.12 to 1 mg/L over this time period. The percent of isolates with a MIC of ≤0.5 mg/L decreased from 85 to 3% (Fig. 4).

#### UK

There was a slight increase in the range of azithromycin MICs between 2001 and 2015 and an increase in modal MIC from 0.12 to 0.25 mg/L [25, 26]. This was associated with a decrease in the proportion of isolates with low MICs (Fig. 4). The midpoint MIC distribution (2003) revealed a MIC distribution that was intermediate between those of 2001 and 2015.

#### USA

Between 1992 and 2017 there was an increase in the modal azithromycin MIC from 0.06 to 0.25 mg/L and an increase in the MIC range from 0.03–0.5 mg/L to 0.03–16 mg/L. The midpoint MIC distribution was from 2013 and was very similar to that of 2017.

#### EUCAST

The EUCAST wild type azithromycin MIC distribution was right shifted compared to the distribution from 1992 in the USA (Fig. 4). The modal MIC and MIC range for EUCAST were both one dilution higher.

### Ceftriaxone

#### Japan

**Fig. 5:**
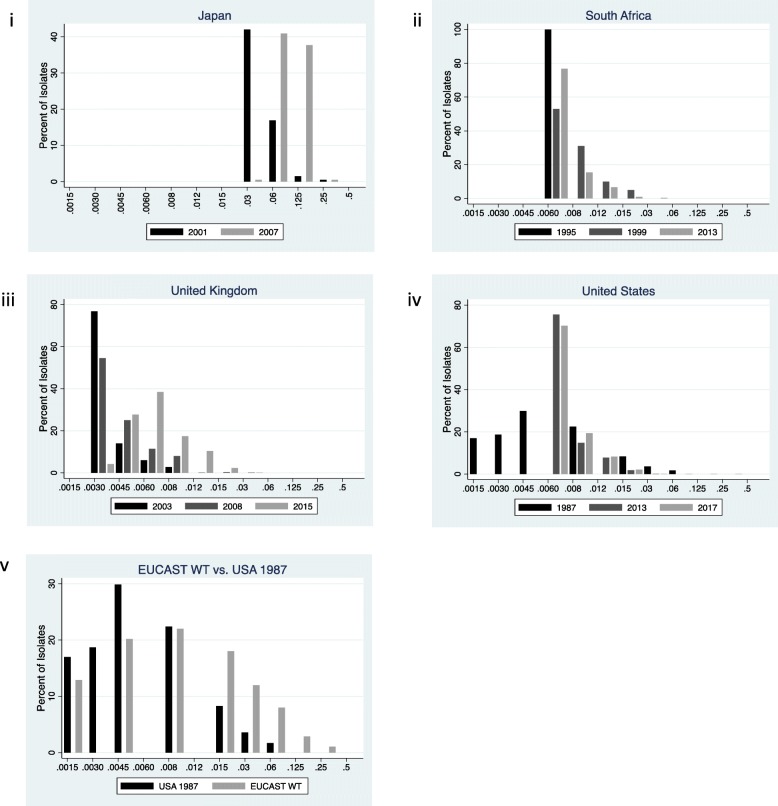
Distribution of *Neisseria gonorrhoeae* ceftriaxone minimum inhibitory concentrations (MIC) distributions for Japan (**i**), South Africa (**ii**), United Kingdom (**iii**) and the United States (**vi**), and EUCAST wild type collection (EUCAST WT) (**v**). All MIC values are reported in mg/L. (The lowest ceftriaxone concentration tested was 0.001 in 1987 and 0.008 mg/L in 2017. The distribution of strains with MIC below 0.008 mg/L can therefore not be compared between these years)

Between 1995 and 2005 there was a large increase in the prevalence of isolates with decreased-susceptibility to ceftriaxone (MIC > 0.125 mg/L) from 0% of 34 isolates in 1995 to 29.1% of 55 isolates in 2003 and 14.7% of 34 isolates in 2005 [19]. A commensurate increase in cefixime resistance (MIC > 0.125 mg/L) was noted from 2.9% in 1995 to 47.3% in 2003 and 29.4% in 2005, in Kanagawa, Japan [19]. These studies did not report MIC distributions and thus this could not be graphically depicted. An unpublished study from the same last author did however provide MIC distributions from 2001 and 2007 and this demonstrated a large decrease in the proportion with ceftriaxone in the lowest MIC categories tested – from 42 to 0.5% for MIC of < 0.03 mg/L (Fig. 5) [37]. This was associated with commensurate increases in higher MICs without an increase in the MIC range. Between 1993/94 and 2013 there was little movement in the MIC range (≤0.001–0.25 to 0.001–0.12, respectively) and MIC50–0.016 and 0.015, respectively [3]. Studies from elsewhere in Japan showed increases in ceftriaxone and cefixime MICs [2].

#### South Africa

In 1995, 100% of isolates were in the lowest MIC category tested (≤0.007 mg/L). By 1999, this had dropped to 53% [22]. These data were obtained using same methodology testing patients attending the same clinics. By 2013 the proportion with MICs of ≤0.007 mg/L had increased again to 77% (Fig. 5) [21].

#### UK

Between 2003 and 2015, the modal MIC increased from < 0.002 to 0.008 mg/L and the highest MIC increased from 0.008 mg/L to 0.3 mg/L [26]. This was associated with a reduction of isolates in the lowest MIC category over this time course (76.8 to 4%; Fig. 5). The MIC distribution for 2008 was not substantially different, though a slight right shift may be present.

#### USA

The lowest ceftriaxone concentration tested was 0.001 in 1987 and 0.008 mg/L in 2017 [31, 34]. The distribution of strains with MIC below 0.008 mg/L can therefore not be compared between these years. In 2017 0.2% of isolates had MICs ≥0.125 mg/L. This proportion was not reported in 1987, when the highest MIC reported was ≥0.06, but the GISP reports note that this proportion has not changed since 2012 [34]. The MIC distribution in 2013 was similar to that in 2017.

#### EUCAST

The EUCAST wild type ceftriaxone MIC distribution was right shifted compared to the distribution from 1987 in the USA (Fig. 5).

## Discussion

Our results provide support for both the bimodal (type A) and the general shift (type B) of MIC distributions over time. The changes in MIC distribution for ceftriaxone fit best with a type B shift where the key change in MIC distribution has been an increase in the prevalence of isolates with higher MICs and a commensurate decrease but not elimination of isolates with low MICs. This is most clearly seen in South Africa where in 1995, 100% of isolates had a MIC of ≤0.007 mg/L, but by 2013 this had been reduced to 77%. In all three countries, where this could be assessed, the gonococci with the lowest MIC assessed were not eradicated over time.

The changes in MIC distribution for benzylpenicillin fit better with a type A shift. In Boston, for example, the pre-1947, pre-penicillin isolates are approximately normally distributed around a MIC of 0.003 mg/L. By 1949, a new sub-population of more resistant isolates has emerged centering on a MIC of 0.015 mg/L (type A, stage 1). By 1987, the USA distribution is approximately normal once again but now centered around a MIC of 0.125 mg/L (type A, stage 2). Only 1.5% of the 1987 isolates had a MIC of ≤0.012 mg/L.

Similarly, in Denmark, the introduction of penicillin is followed by a new bimodal distribution (type A, stage 1). The earliest benzylpenicillin MIC distributions from South Africa (1976) and the UK (1957) are from the penicillin period and demonstrate bimodal distributions. The later distributions from these countries are right shifted compared to the earlier period and in the case of South Africa, exhibit a new bimodal distribution including a new population centered on a MIC of 8 mg/L (type A, stage 3). The earliest data from Japan (1968) do not demonstrate a bimodal peak but a unimodal distribution centered around a high MIC of 0.5 mg/L. By 2009, similar to South Africa, bimodality has emerged as a result of the emergence of a new population with MICs around 1 mg/L (type A, stage 3).

The results for azithromycin are more mixed. South Africa’s trajectory fits best with type A, stage 2 – the whole MIC distribution is right shifted. In the UK and USA on the other hand, the distribution has been stretched to the right and thus best characterized as type B shifting.

The different trajectories of azithromycin, ceftriaxone and benzylpenicillin may be due to the different length of time they were observed for. This hypothesis is compatible with the fact that benzylpenicillin which has the longest follow-up data available exhibited the most marked changes in MIC distribution. As longer follow-up data become available for ceftriaxone and azithromycin they may follow a similar trajectory to benzylpenicillin. Formally defining type A and B shifts was beyond the scope of this analysis. We therefore acknowledge that our classification of MICs shifts into these types will remain open to debate until larger datasets are analyzed that enables a more formal classification of how MICs change over time per species. A further considerable limitation of these analyses is that changes in MIC distributions between studies may reflect differences in ascertainment of susceptibility. An example of this is a change of supplier of the agar used for susceptibility testing in the UK’s GRASP in 2015, which was shown to lead to slight increases in MICs for benzylpenicillin, ceftriaxone and azithromycin [38]. These limitations would not however apply to the benzylpenicillin analyses from Denmark where the isolates from both periods were tested in parallel using the same methodology. Further limitations include the fact that we used the susceptibility data as they were reported and did not assess if publication or selection biases were present or absent. In particular we did not assess if changes in the population sampled may have influenced the results. For example, if subpopulations with higher rates of AMR were more likely to be included in the later dates this may lead to a right shifting of MIC distributions. The potential for this bias is particularly relevant in three of our comparisons: 1. Benzylpenicillin, Japan 1968 versus 2009–2010. In 2009 the samples were form men attending an STI clinic whereas the samples from 1968 were from navy personnel who may have been more likely to have obtained their infections from sex workers who have been shown to have more resistant strains in some studies [39], 2. Benzylpenicillin, the USA 1945–1949 versus 1987-onwards. The 1940s isolates were from men and women versus the post 1986 isolates which were all from men. 3. Azithromycin, the UK, 2001 versus 2015. The proportion of samples that were from MSM was considerably higher in 2015 which may been responsible for the right shifting of the MIC distribution [39, 40]. We were also unable to assess if unreported quality issues may have affected the accuracy of the data. Other limitations include that our literature search may have missed published reports, particularly in the early period when the indexing of articles was suboptimal. A further problem was that our analysis was limited to three antimicrobials in four countries, which limits generalizability. Finally, our analysis is descriptive and not quantitative.

Our findings from the pre-penicillin period are however congruent with those from 16 other studies which found that gonococci in this period were highly susceptible to penicillin (reviewed in [16]). Likewise, our findings of a bimodal penicillin MIC distribution emerging soon after significant penicillin exposure are similar to those in multiple populations around the world [41–43]. There is also evidence from other populations not included in our review of the emergence of a second bimodal peak in the penicillin MIC distribution in more recent decades [24, 44]. These studies thus provide some support for our finding that reductions in susceptibility to penicillin, at least in certain populations, are best characterized by type A shifts that evolve through up to 3 stages. By the third stage, benzylpenicillin MIC distributions such as those from Japan and South Africa were so right-shifted that no or very few isolates have MICs ≤0.03 mg/L. Their distribution no longer overlaps with that of the pre-penicillin population. How can we explain this?

### What mechanisms underpin the decline/extinction of the lowest MIC populations?

We propose that 3 mechanisms may be relevant: 1) The zero-sum nature of changes in distributions means that an increase in the proportion with high MICs necessarily entails a commensurate decrease in the proportion with lower MICs; 2) Sustained antimicrobial exposure may have led to a selective sweep of *N. gonorrhoeae* whereby highly sensitive strains were eliminated from the gene pool/local pangenome; 3) Sustained antimicrobial pressure may have selected for antimicrobial resistance in commensal bacteria, including commensal *Neisseria* spp. If this change is profound enough and there is sufficient DNA exchange with these commensals then this could result in the decline or elimination of low MIC strains in both commensal and pathogenic species.

The first mechanism is able to explain a decline but not the elimination of low MIC isolates. Support for the second mechanism comes from studies that have found associations between the intensity of antimicrobial consumption in the general population and homologous gonococcal AMR [6, 9]. Furthermore, studies have found that particular gonococcal genogroups can emerge and disseminate rapidly displacing other genogroups. For example, genogroup 1407, which is strongly associated with reduced cephalosporin susceptibility, emerged explosively in Europe around 2007 and rapidly displaced other more susceptible genogroups [45]. In support of the third mechanism, recent receipt of ciprofloxacin, ceftriaxone or cefixime has been established as an independent risk factor for homologous AMR in oropharyngeal commensal *Neisseria* [8]. The relative contributions of mechanisms 2 and 3 is likely to vary between different classes of antimicrobials. For example, horizontal gene transfer of resistance conferring genes from commensal *Neisseria* has been shown to have played an important role in cephalosporin and macrolide resistance but not for fluoroquinolone resistance [46].

## Conclusions

The major relevance of this study is showing the utility of longer-term analyses of MIC distribution. Contemporary analyses of gonococcal AMR are frequently limited to the last decade or so [6, 9, 47]. These analyses will likely miss changes in MIC distribution that occur slowly over longer periods. In other fields (mainly environmental conservation) this short-term focus has led to what has been termed the shifting baseline syndrome, where in the absence of sufficient knowledge of historical conditions, members of each new generation accept the situation in which they are raised as normal [48]. An important consequence of this syndrome is that it increases an individual’s tolerance of man-made environmental damage.

It is important to remember that the emergence of AMR in *N. gonorrhoeae* is not inevitable. Populations in countries such as the Netherlands with low rates of consumption of macrolides, quinolones and cephalosporins have correspondingly lower rates of gonococcal AMR than high consumption countries [6]. The treatment of choice for gonorrhoea in the rural part of the Northern Territories, Australia, remains penicillin, because the prevalence of penicillin resistance here is still less than 1% [49].

These findings indicate that it is possible to contain the emergence of gonococcal AMR.

The fact that a unimodal distribution can re-emerge in stage 2 of a type A shift may compound the shifting baseline problem. Observers looking at a stage 2 MIC distribution in isolation may conclude that this represents a wild type distribution. Indeed, contemporary datasets are favored in the construction of epidemiological cutoffs (ECOFFs) for MICs [15]. If one analyzes EUCAST’s ‘Antimicrobial wild type distributions of microorganisms’ gonococcal dataset with the EUCAST recommended software “ECOFFinder”, this generates an ECOFF for benzylpenicillin of 8 mg/L [15]. Gonococcal isolates with MICs of over 1 have a high likelihood of non-response to benzylpenicillin therapy [42] which illustrates why it is not meaningful to try to find penicillin ECOFFs for contemporary gonococcal populations that have experienced right shifting of their MICs. Evaluating ECOFFs from the pre-penicillin populations would however be more appropriate. Following our historical methodology, the EUCAST ECOFF MIC distribution would be characterized as having undergone a type A, stage 3 shift. Likely because more recent datasets are favored in the EUCAST dataset, the older MIC distributions, such as those from 1945 (Fig. 3), are not included in this distribution.

Further studies could analyze what mechanisms underpin the elimination of highly susceptible strains, what the correlates are of populations that have undergone a stage 3 as opposed to a stage 1 or 2, type A shift in penicillin/other antimicrobial AMR? Can this all be explained by differential antimicrobial exposure? If so are there thresholds of consumption that populations should aim to not exceed [10]? Which gonococcal genotypes/genogroups have been driven extinct in populations with stage 2 and 3 shifts? Is this mirrored by a similar extinction in susceptible strains in commensal species? Finally, it may be useful to investigate if this process has played a role in the reduction in diversity of the gut microbiome in Western populations [50, 51].

## Data Availability

The data we used are available from the authors on request.

## Abbreviations

AMR: Antimicrobial resistance
GISP: Gonococcal isolate surveillance project
GRASP: Gonococcal resistance to antimicrobials surveillance programme
GUM: Genitourinary medicine clinic
MSM: Men who have sex with men
STD: Sexually transmitted diseases
UK: United Kingdom
USA: United States of America
